# Draft Genome Sequences of Three *Salmonella enterica* Serotype Agona Strains from China

**DOI:** 10.1128/genomeA.00203-12

**Published:** 2013-02-21

**Authors:** Jianmin Zhang, Guojie Cao, Xuebin Xu, Huimin Jin, Xiaowei Yang, Marc Allard, Eric Brown, Jianghong Meng

**Affiliations:** a Shanghai Jiao Tong University, Shanghai, China; b Department of Nutrition and Food Science, University of Maryland, College Park, Maryland, USA; c Joint Institute for Food Safety and Applied Nutrition (JIFSAN), University of Maryland, College Park, Maryland, USA; d Shanghai Municipal Center for Disease Control and Prevention, Shanghai, China; e Division of Microbiology, Center for Food Safety and Applied Nutrition, Food and Drug Administration, College Park, Maryland, USA

## Abstract

Salmonellosis has been one of the major contributors to the global public health burden. *Salmonella enterica* serotype Agona has ranked among the top 10 and top 20 most frequent *Salmonella* serotypes isolated from human sources in China and the United States, respectively. We report draft genomes of three *S.* Agona strains from China.

## GENOME ANNOUNCEMENT

Nontyphoidal *Salmonella* has been a significant public health burden globally, causing 93.8 million cases of gastroenteritis and 155,000 deaths annually (1). Nontyphoidal *Salmonella* serotypes have caused over one million illnesses (2) and several billion dollars in economic losses in the United States each year (3). *Salmonella enterica* serotype Agona is ranked as the 10th most frequent nontyphoidal *Salmonella* serotype isolated from infected humans in China (4) and ranked among the top 20 most frequent *Salmonella* serotypes in the United States (5). *S.* Agona is an important zoonotic (6) and food-borne (7) pathogen, causing serious human illness (7). It was isolated initially from cattle in Ghana (8) and has emerged as a significant pathogen on a global level (9, 10). Moreover, *S.* Agona was thought to be highly associated with a multidrug resistance gene cluster (11). We selected three multidrug-resistant *S.* Agona strains from China for whole-genome sequencing analysis.

Currently, there are 34 completed genomes and 163 draft genomes of *Salmonella* that have been deposited in GenBank. However, there is only one genome of *S.* Agona available (*S.* Agona SL483). In the present report, we announce the availability of three draft genomes of *S.* Agona strains: SH11G1113 (human stool, 2011, China), SH08SF124 (cattle feces, 2008, China), and SH10G094 (human stool, 2010, China). The availability of genome sequences for these Asian strains will provide unique insights and better understanding of the evolutionary history and pathogenicity of *S.* Agona.

The three *S.* Agona strains were sequenced using the MiSeq personal sequencer (Illumina, San Diego, CA) to obtain 30 to 41× coverage high-quality draft genomes. Genomic DNA from each strain was isolated from overnight culture. Genomic data were assembled with the Celera assembler (v 7.0). The data of each draft genome follow: SH11G1113 (78 contigs, 4,856,197 bp, and 90,606 bp N_50_ contig size), SH08SF124 (63 contigs, 4,889,188 bp, and 184,703 bp N_50_ contig size), and SH10G094 (66 contigs, 4,811,275 bp, and 197,357 N_50_ contig size). Sequences were annotated with the NCBI Prokaryotic Genomes Automatic Annotation Pipeline (12). A total of 4,611 (SH11G1113), 4,677 (SH08SF124), and 4,583 (SH10G094) genes were identified.

A detailed report of comparative genomic and phylogenetic analysis of the three draft genomes will be included in a future publication.

### Nucleotide sequence accession numbers.

The draft genome sequences of these three *S.* Agona strains are available in GenBank under accession nos. ANOS00000000, ANOT00000000, and ANOU00000000. The versions described in this paper are the first versions: ANOS01000000, ANOT01000000, and ANOU01000000.
